# An effective classification framework for brain-computer interface system design based on combining of fNIRS and EEG signals

**DOI:** 10.7717/peerj-cs.537

**Published:** 2021-05-06

**Authors:** Adi Alhudhaif

**Affiliations:** Department of Computer Science, College of Computer Engineering and Sciences in Al-Kharj, Prince Sattam bin Abdulaziz University, Al-Kharj, Saudi Arabia

**Keywords:** Near-infrared spectroscopy, Brain-computer interfaces, Feature weighting, Motor imaginary, EEG

## Abstract

**Background:**

The brain-computer interface (BCI) is a relatively new but highly promising special field that is actively used in basic neuroscience. BCI includes interfaces for human-computer communication based directly on neural activity concerning mental processes. Fundamental BCI components consist of different units. In the first stage, the EEG and NIRS signals obtained from the individuals are preprocessed, and the signals are brought to a certain standard.

**Methods:**

In order to realize proposed framework, a dataset containing Motor Imaginary and Mental Activity tasks are prepared with Electroencephalography (EEG) and Near-Infrared Spectroscopy (NIRS) signal. First of all, HbO and HbR curves are obtained from NIRS signals. Hbo, HbR, HbO+HbR, EEG, EEG+HbO and EEG+HbR features tables are created with the features obtained by using HbO, HbR, and EEG signals, and feature weighted is carried out with the k-Means clustering centers based attribute weighting method (KMCC-based) and the k-Means clustering centers difference based attribute weighting method (KMCCD-based). Linear Discriminant Analysis (LDA), Support Vector Machine (SVM), and k-Nearest Neighbors algorithm (kNN) classifiers are used to see the classifier differences in the study.

**Results:**

As a result of this study, an accuracy rate of 99.7% (with kNN classifier and KMCCD-based weighting) is obtained in the data set of Motor Imaginary. Similarly, an accuracy rate of 99.9% (with SVM and kNN classifier and KMCCD-based weighting) is obtained in the Mental Activity dataset. The weighting method is used to increase the classification accuracy, and it has been shown that it will contribute to the classification of EEG and NIRS BCI systems. The results show that the proposed method increases classifiers’ performance, offering less processing power and ease of application. In the future, studies could be carried out by combining the k-Means clustering center-based weighted hybrid BCI method with deep learning architectures. Further improved classifier performances can be achieved by combining both systems.

## Introduction

**Figure 1:**
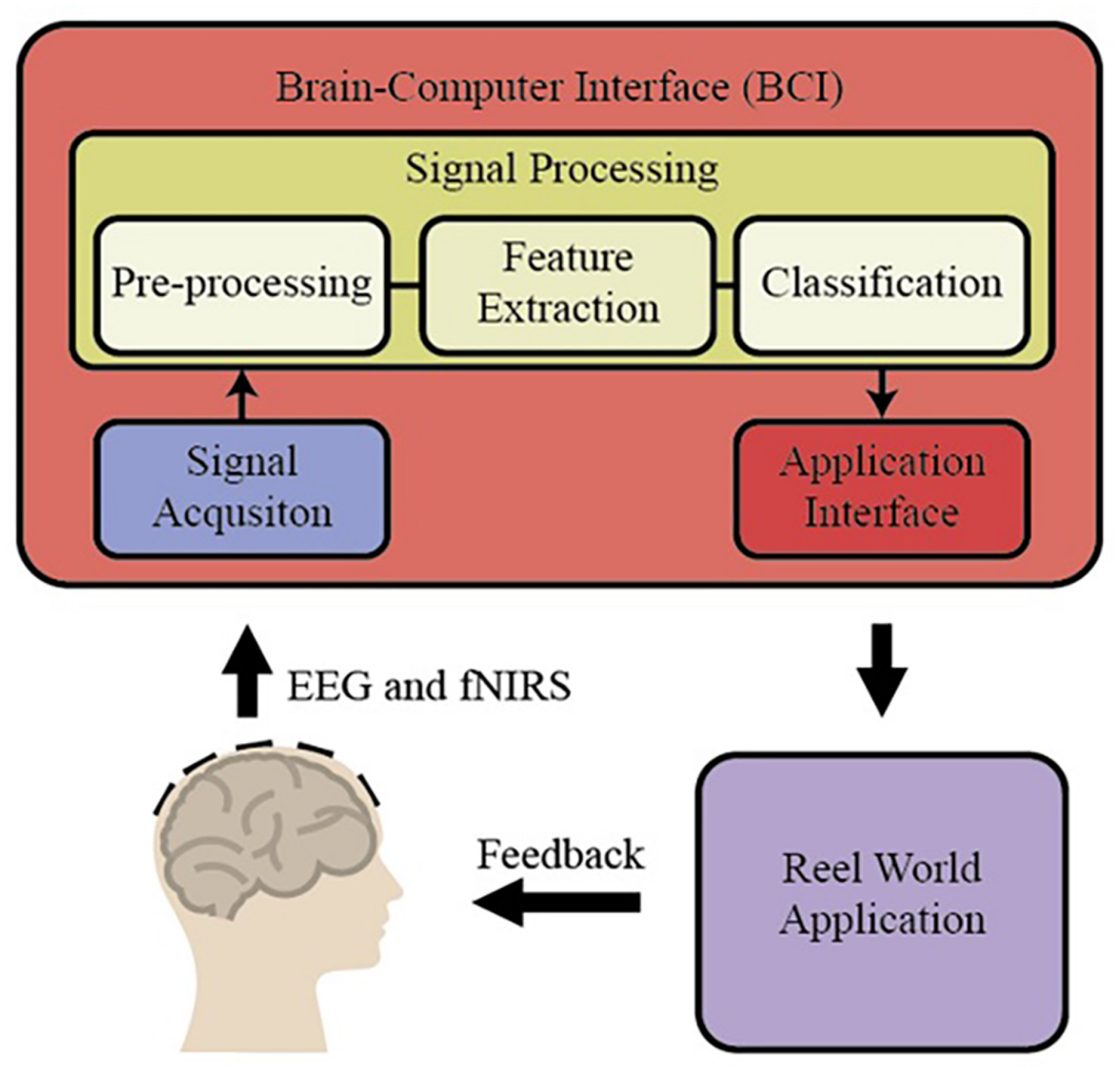
The essential trading components of BCI system.

BCI is a special area of recent applications in basic neuroscience. BCI includes interfaces for human-computer communication based directly on neural activity concerning mental processes. Some of the BCI research in the literature focuses only on developing direct communication and control methods based on neural activity in the brain. Some of the brain's neural sensors with sensor data for vision or sensory values are collected by artificial sensors over the methods that directly acquire the systems and eliminate the non-functional sensory organs’ deficiencies (https://dergipark.org.tr/tr/download/article-file/340760). These essential trading components of BCI are shown in Fig. 1 (https://www.researchgate.net/publication/267792090_4_Human_Brain-Computer_Interface).

Basic BCI components consist of different units. In the first stage, the EEG and fNIRS signals obtained from the individuals are preprocessed (filtering and normalization), and the signals are brought to specific standards. Then, different characteristic features are extracted from the EEG and fNIRS signals on the time and frequency axis using hand-crafted or automatic methods. In BCI applications, these extracted features for the desired purpose are applied to the classification algorithm, and thus high performance is targeted. By applying the obtained classification result to the real world, it is reached the final result. There are different methods to measure brain activity. Techniques that measure brain activity, without intervention, that is, without entering the tissue: EEG, functional magnetic resonance imaging (fMRI), and NIRS (http://noroblog.net/2019/01/06/beyin-bilgisayar-arayuzunden-beyinler-arasi-iletisime/). BCIs transform the brain activities taken from the individual into action or writing through this application. For this purpose, it uses EEG signals, which is one of the methods that visualize brain activities. The obtained EEG signals are transformed into meaningful information by using machine learning methods through computers. EEG signal acquisition is carried out through electrodes, and a large number of electrodes are used for this process. The excess number of electrodes increases the required capacity of the electronic and computer equipment used. This situation causes an increase in both the financial burden and the processing load. Therefore, the systems’ physical dimensions increase and the cost exceeds the values accessible to everyone. BCI systems are also tested for applications that require the assessment of mental states such as attention level, stress, workload. This is called passive BCI. One of the tested application areas is activities such as driving a car or airplane where safety is critical and human error can lead to serious consequences. A serious amount of research focuses on the effect of sleepiness and fatigue on EEG waves in their studies on drivers. Some BCI works are using EEG, fNIRS, and the combination of EEG and fNIRS signals in the literature. Among them, some works have been explained briefly in the following. Shin et al. (2017) proposed a new open-access dataset on brain-computer design with EEG and fNIRS signals and obtained new results on BCI by using many different signal processing and machine learning methods. In another study, Chiarelli et al. (2018) combined EEG and fNIRS signals with the deep learning algorithm, designed a new BCI system, and applied it to the motor imagery classification problem. During the classification stage, they used LDA, SVM, and deep neural network (DNN). Shin, Kwon & Im, (2018) proposed a new hybrid model to classify brain function during mental arithmetic, motor imagery, and Idle State. They used a shrinkage linear discriminant analysis (sLDA) classification algorithm to classify the features obtained from EEG and fNIRS signals. They achieved classification accuracies of 76.1 ± 12.8%, 64.1 ± 9.7%, and 82.2 ± 10.2% with EEG-BCI, NIRS-BCI, and hBCI models (Shin, Kwon & Im, 2018). In another study, Janani et al. (2020) used fNIRS signals to classify engine imagery states as a BCI application. They used SVM, multilayer perceptron (MLP) neural network, and convolutional neural network (CNN) as the classification method in their study and achieved an accuracy rate of 72.35 ± 4.4% with the CNN method (Janani et al., 2020). Aydin (2020) proposed a new hybrid machine learning model using fNIRS signals to classify mental arithmetic and motor imagery states. To reduce the number of features obtained from fNIRS signals, the number of features is reduced with sequential feature selection and ReliefF feature selection methods, and they achieved classification success for each case with three different classification algorithms, including Linear discriminant analysis, k nearest neighborhood, and support vector machines (Aydin, 2020).

Instead of creating a heavy computational load as in the studies suggested in the literature, this study proposes a new and efficient machine learning model and applies it to the problem of motor imagery and mental activity classification. The proposed framework uses both EEG and fNIRS signals together. It can be summarized as follows: From the oxy-hemoglobin (HbO) and deoxy-hemoglobin (HbR) curves obtained by fNIRS signals and EEG signals, features are obtained singularly or combined. Secondly, the obtained singular and hybrid features are weighted according to the classes. The classification process's contribution with LDA, SVM, and kNN classifiers and hybrid signals and weighting to the classification performance is examined in the last step.

The methods used in the study are preferred for reasons such as ease of application and ease of processing. The primary purpose is to reveal the power of weighting algorithms, which recommend for the first time in Mental Activity and Motor Imaging studies, rather than to compare classifiers’ performance. There are many advantages of deep learning algorithms in BCI systems (Zhang et al., 2018; Zhang et al., 2019). However, it is not possible to interfere with the inner workings of deep learning architectures. Therefore, it will be difficult for us to distinguish whether the results obtained after the classification come from the method proposed or the power of deep learning. Thus, traditional features and classifiers are used to compare the literature and apply it to the suggested method.

The novelties of the study can be listed as follows:

- The features of the singular and combined HbO, HbR, and EEG signals are weighted with a fast-weighting algorithm and k-means clustering-based weighting algorithms.
- An improvement in classifier performance is achieved with the base classifiers without a negative effect on processing speed.

## Materials & methods

### EEG+NIRS single-trial classification dataset

“Open Access Dataset for EEG+NIRS Single-Trial Classification” is used to reveal the proposed framework’s performance in the study (Shin et al., 2017; Blankertz et al., 2010). This dataset consists of NIRS and EEG signals, including mental activity (MA) and motor imaginary (MI), two separate tasks. A total of 29 users (15 females, 14 males; 28 right hands, 1 left hand) participated in the study.

**Figure 2:**
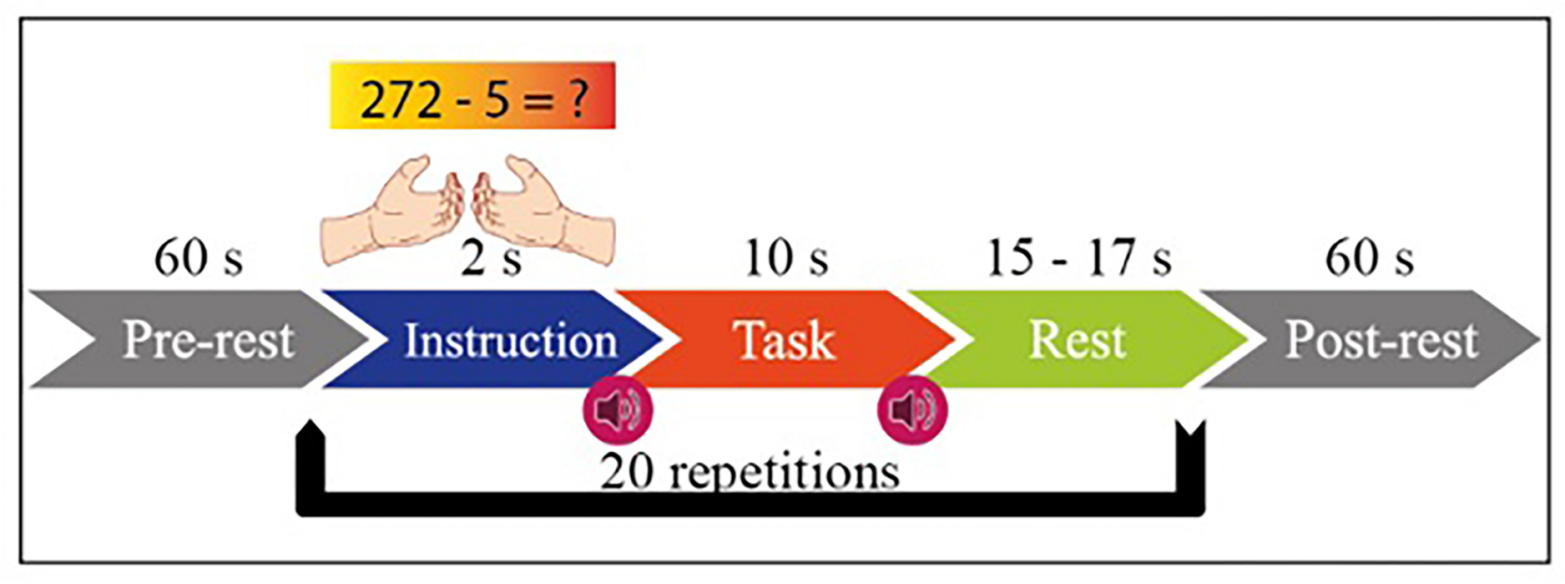
Experimental paradigm for MI and MA in one session.

MI has two functions in itself, right hand and left hand. MA includes mental processing and resting-state tasks within itself. The experimental setup is designed with the instructions given to the subject sitting 1.6 m in front of the 50-inch screen. The paradigm of the experiment is given in Fig. 2. Both tasks started with one-minute rest before the experiment. Then, 2 s of visual information about the task, 10 s of task execution, and 15–17 s of rest after the task are given.

**Figure 3:**
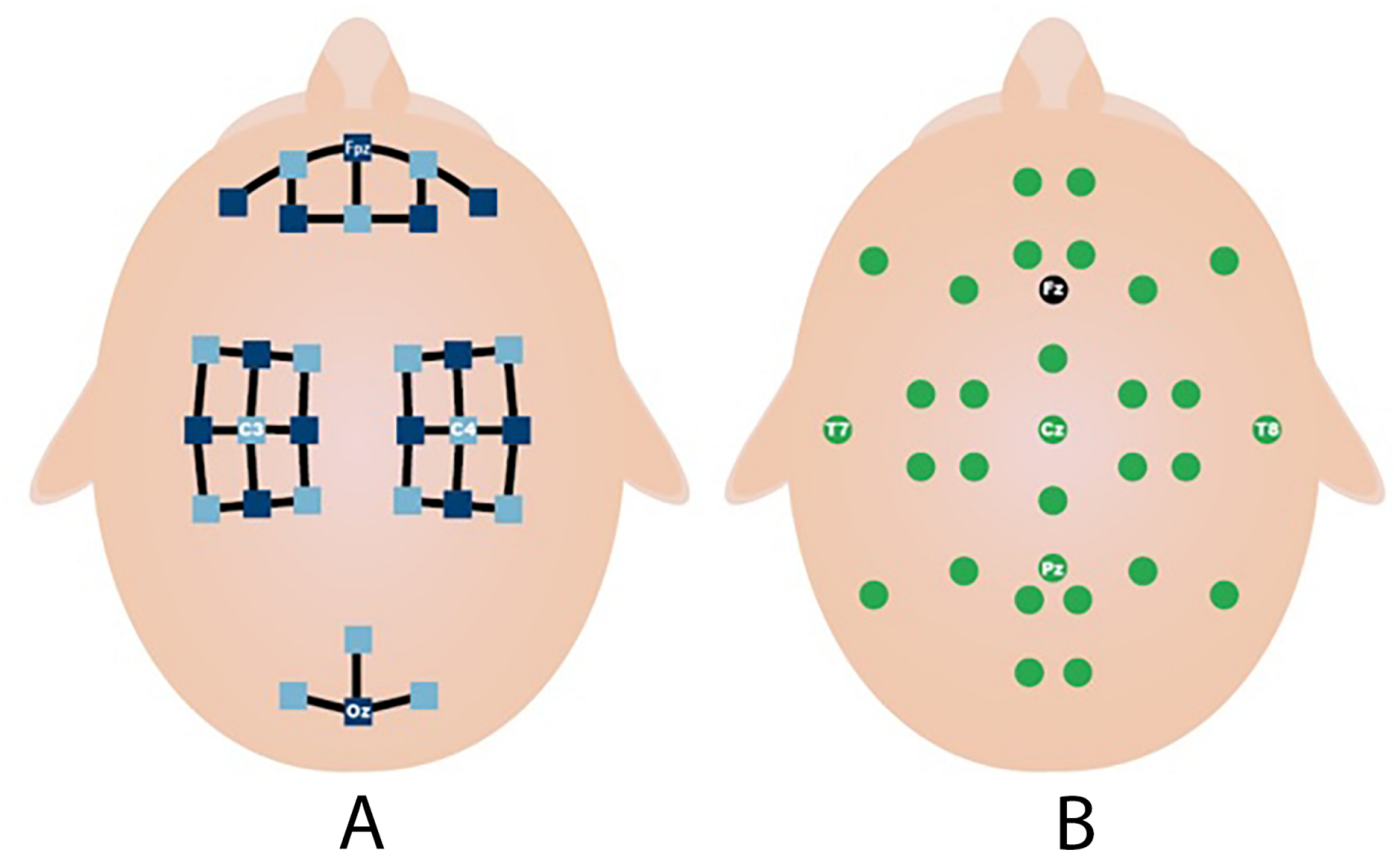
Electrodes placement (A) fNIRS sources (dark blue) and detector (light blue) channels. (B) EEG electrodes and ground (black) (Shin et al., 2017).

This process is repeated 20 times in each session. MI and MA tasks are recorded sequentially and in 3 sessions. As shown in Fig. 3A, fNIRS recordings are taken with 36 physiological channels produced using 14 sources and 16 detectors. The recording is performed with a sampling frequency of 12.5 Hz. The recordings are then downsampled at 10 Hz. Figure 3B shows that 30 EEG electrodes placed according to the international 10-5 system are given. The signals are collected with a 1,000 Hz sampling frequency and then downsampled at 200 Hz (Shin et al., 2017).

### Preprocessing of the EEG and fNIRS Signals

**Figure 4:**
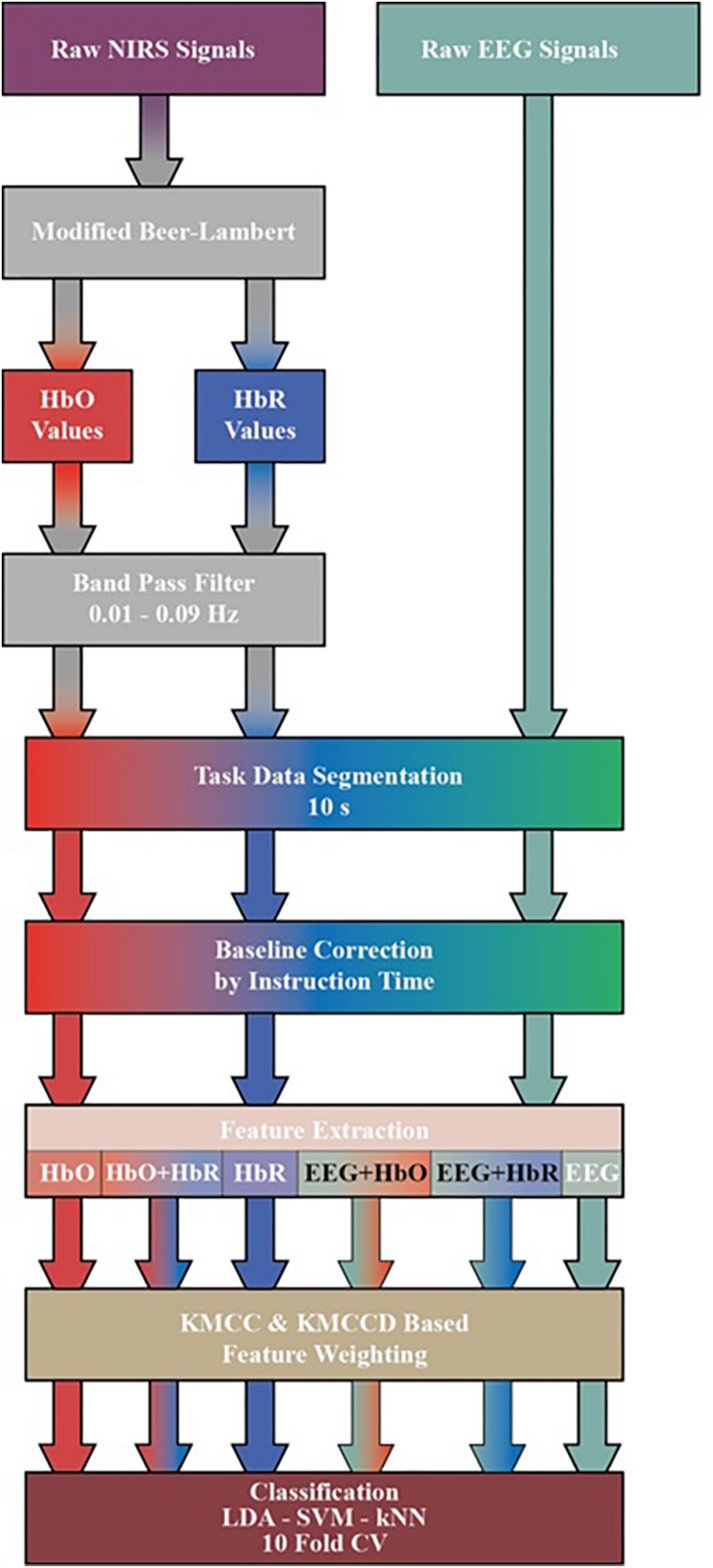
Diagram of proposed processing and classification method hybrid fNIRS and EEG signals.

Raw fNIRS and EEG signals obtained from the dataset are subjected to a series of processes before classification. The transactions performed are shown in Fig. 4. First, by applying the Modified Beer-Lambert law given in (1) to fNIRS signals, the concentration changes of oxyhemoglobin (HbO) and deoxyhemoglobin (HbR) are calculated (Chiarelli et al., 2018; Trakoolwilaiwan et al., 2017; Shin & Jeong, 2014).

(1) $$\left[\begin{array}{c} \Delta \left[HbO\right]\\ \Delta \left[HbR\right] \end{array}\right]=\frac{1}{\rho }{\left[\begin{array}{cc} \varepsilon_{O_{2}Hb}\left(\lambda_{1}\right).DPF\left(\lambda_{1}\right) & \varepsilon_{HHb}\left(\lambda_{1}\right).DPF\left(\lambda_{1}\right)\\ \varepsilon_{O_{2}Hb}\left(\lambda_{2}\right).DPF\left(\lambda_{2}\right) & \varepsilon_{HHb}\left(\lambda_{2}\right).DPF\left(\lambda_{2}\right) \end{array}\right]}^{-1}\times \;\left[\begin{array}{c} OD_{\lambda_{1}}\\ OD_{\lambda_{1}} \end{array}\right]$$

The HbO and HbR curves are filtered with a 3rd order 0.01–0.09 Hz Butterworth bandpass filter. Processes after this step are common for both fNIRS and EEG signals. Three sessions and 20 repetitions in each session are segmented from the moment stimulation began (0 s) to the moment it ended (10 s). The obtained signals are subjected to the baseline correction process with the average of the signal generated at the instruction stage before stimulation (−2 s to 0 s).

### Feature extraction from the EEG and fNIRS Signals

The representation ability of hand-crafted features is known, especially in the analysis of complex signals. Mean, maximum, slope, variance, skewness, kurtosis, and median features frequently used in fNIRS signals are used in the literature (Aydin, 2020; Chiarelli et al., 2018). The feature combination of the proposed framework is as follows:

**(1) Mean: **It is the average amplitude value of each epoch signal. It is calculated by Eq. (2). Here μ mean value, N total data points, $X_{k}$ attribute of the signal to be calculated data point.

(2) $$\mu =\frac{1}{N}\overset{N}{\underset{k=1}{\sum }}X_{k}$$

**(2) Maximum:** It is the highest amplitude value of each epoch signal.
**(3) Slope:** It is the average of the slopes in a defined time window over the entire signal.
**(4) Variance:** It is the value showing the distance of the distribution from the mean in the data. Where V is variance, N total data point, μ arithmetic mean and $X_{k}$ attribute of the signal to be calculated data point.

(3) $$V=\frac{1}{N-1}\overset{N}{\underset{k=1}{\sum }}{\left|X_{k}-\mu \right|}^{2}$$

**(5) Skewness**: It is the value that gives the degree of non-symmetry of a distribution. X is data, μ is the mean, σ is the standard deviation, and E represents the expected value.

(4) $$s=\frac{E{\left(x-\mu \right)}^{3}}{\sigma^{3}}$$

**(6) Kurtosis:** It is the value that gives the sharpness or flatness of the curve. Where x, μ, σ, and E same value in skewness.

(5) $$s=\frac{E{\left(x-\mu \right)}^{4}}{\sigma^{4}}$$

**(7) Median:** It is the value in the middle of the sorted data.

These seven features are calculated for each channel. Then, the feature space is created by adding each channel side by side. Six feature matrices are created for MI and MA after preprocessing. These matrices are HbO, HbR and Hbo+HbR obtained from fNIRS signals, EEG obtained from EEG signals, and their combination EEG+HbO and EEG+HbR.

As stated under the title of feature extraction in the study, seven features have been extracted for each channel. Since fNIRS signals are 36 channels, 36 * 7 = 252 features are obtained from HbO and HbR values obtained after Beer-Lambert transformation. The attribute matrix with tags has 253 columns. Similarly, 32 * 7 = 224 features are obtained from 32 channel EEG signals. Together with the tag column, an attribute matrix of 225 columns is obtained. In the hybrid studies, 476 features are obtained from 252 + 224 features for EEG+HbO and EEG+HbR data, while 252 + 252 = 504 features are obtained from HbO+HbR data. Tag vectors are then added to these. The number of observations made is; The data belonging to each user is divided into 10-s epochs from the start of the task. In this setup, 20 observations for each session and 60 observations in a total of 3 sessions are obtained. When 29 users are combined, 60 * 29 = 1740 observations are obtained. In summary;

- HbO and HbR feature vectors (1740, 253),
- HbO+HbR hybrid feature vectors (1740, 505),
- EEG attribute vector (1740, 225),
- The EEG+HbO and EEG+HbR hybrid feature vectors are of the size (1740, 476).

### Feature (attribute) weighting algorithm

The main purpose of the feature weighting process is to transform nonlinearly separable data into a linearly separable form. In the study, two different k-means clustering methods are used for the weighting of the features. K-Means clustering methods pseudo-code is given in Table 1 (Polat & Durduran, 2012).

**Table 1:** Pseudo code for k-means clustering method.

Step	Procedure
Step 1	Choose k=2 initial cluster centers $z_{1},\;z_{2}$ randomly from the *n* points$\left\{x_{1},\;x_{1},\;\ldots ,\;x_{n}\right\}$
Step 2	Define point $x_{i}$, i=1,2,…,n the cluster $C_{j}$, j=1,2 If $x_{i}-z_{1}<x_{i}-z_{2}$,
Step 3	Calculate new cluster centers; $z_{j}^{new}=\frac{1}{n_{j}}\underset{x_{i}\in C_{j}}{\sum }x_{i}$ j=1,2
Step 4	If $z_{j}^{new}-z_{j}<\varepsilon$, j=1,2, then execute. Otherwise repeat from step 2 to 4

### k-means clustering centers based attribute weighting method (KMCC-based)

The center of each feature set is found by k-means clustering (KMC), and then the ratio of the feature means to the cluster center is calculated. The pseudo-code for the method is given in Table 2 (Polat & Durduran, 2012). Where i is class number, j is features number, $c_{i}$ are feature matrixes for two-class, $z_{i}$ are cluster centers for two feature matrixes, $\mu_{i,j}$ are the mean value of features for two-class, $w_{i,j}$ are weight values of features for two class and $data_{weighted}$ is KMCC-based weighted data.

**Table 2:** Pseudo code for KMCC-based method.

Step	Procedure
Step 1	Load features matrix and separate by class $c_{i}$, i=1,2
Step 2	Calculate the $z_{i}$ using Table 1, i=1,2
Step 3	Calculate $\mu_{i,j}$ value of features for each class, i=1,2, j=1,2,…,*n*
Step 4	Calculate $w_{i,j}$ values of features for each class $w_{i,j}=\frac{u_{i,j}}{z_{i}}$, i=1,2, j=1,2,…,*n*
Step 5	Calculate weighted data $data_{weighted,i}=c_{i,j}\times w_{i,j}$, i=1,2, j=1,2,…,*n*

### Means clustering centers difference based attribute weighting method (KMCCD-based)

In this method, the center of each feature set is found by KMC, and then the distance of each data point to the cluster center is calculated. The ratio of the mean of these distances to the cluster centers gives the weight value for each feature. The pseudo-code for the method is given in Table 3 (Polat, 2018). Where i is class number, j is features number, $c_{i}$ are feature matrixes for two-class, $z_{i}$ are cluster centers for two feature matrixes, $d_{i,j}$ are a distance of each data point to the cluster center, $\mu_{i,j}$ are the mean value of distances for two-class, $w_{i,j}$ are weight values of features for two class and $data_{weighted}$ is KMCC-based weighted data.

**Table 3:** Pseudo code for KMCCD-based method.

Step	Procedure
Step 1	Load features matrix and separate by class $c_{i}$, i=1,2
Step 2	Calculate the $z_{i}$ using Table 1, i=1,2
Step 3	Calculate the $d_{i,j}$ of each data point to the cluster center $d_{i,j}=c_{i,j}-z_{i}$, i=1,2, j=1,2,…,*n*
Step 4	Calculate $\mu_{i,j}$ value of distances for each class, i=1,2, j=1,2,…,*n*
Step 5	Calculate $w_{i,j}$ values of features for each class $w_{i,j}=\frac{u_{i,j}}{z_{i}}$, i=1,2, j=1,2,…,*n*
Step 6	Calculate weighted data $data_{weighted,i}=c_{i,j}\times w_{i,j}$, i=1,2, j=1,2,…,*n*

### Classifier algorithms

In this section, LDA, SVM, and kNN classifiers are used to observe the effect of classifiers’ performance.

LDA searches for a vector that best separates data points. It creates a linear combination that gives the most significant mean differences according to the classes entered. In this classifier, a primary scoring function is defined, and the coefficients that will maximize this score are sought (Arican & Polat, 2019; Filho et al., 2014; Parah et al., 2020; Ohata et al., 2021).

SVM is a machine learning method recommended for classification problems in datasets where patterns between variables are unknown. SVM is based on statistical learning theory and structural risk minimization. For classification, it is possible to separate the two groups by drawing a boundary between two groups on a plane. The place where this border will be drawn should be the furthest from the members of both groups. Here SVM determines how this border will be drawn. SVMs are classifiers that do not take any parameters (nonparametric). There is no prior knowledge or assumption about the distribution. Inputs and outputs are matched in training sets. Decision functions that will classify the input variable in test sets and new data sets are obtained through the peers (Costantini et al., 2009; Parah et al., 2020; Ohata et al., 2021; Dourado et al., 2021).

kNN is one of the algorithms used for classification in supervised learning. It is considered to be the simplest machine learning algorithm. In model recognition, the nearest neighbor algorithm (kNN) is a nonparametric method used for classification. With kNN, basically, the closest points to the new point are searched. *k* represents the amount of the closest neighbors of the unknown point. The quantity k of the algorithm (*k* = 1 in this study) is chosen to predict the results (Şahan et al., 2007; Filho et al., 2014).

## Results

Classifier performances in the study are evaluated by the accuracy rate obtained from the confusion matrix. The accuracy (ACC) value is obtained from the confusion matrix by (6). The sensitivity (Sens) calculates the correct estimation rate of the positive class by (7). FPR gives the false estimation rate of the negative class by (8). Precision (PRC) calculates how many of the positive predictions are true positive by (9) (Arican & Polat, 2019).

(6) $$ACC\;\%=\frac{TP+FP}{TP+TN+FP+FN}\times 100$$

(7) $$Sens=\;\frac{TP}{TP+FN}$$

(8) $$FPR=\frac{FP}{TP+TN}$$

(9) $$PRC=\;\frac{TP}{TP+FP}$$

Kappa coefficient is a statistical method that measures the reliability of the comparative agreement between two evaluators, and this coefficient is calculated by (10)–(13) (Cohen, 1960). Here p1 is the probability that a tag randomly selected from the data set is positive, and p2 is the probability that the classifier finds it positive.

(10) $$Kappa\;=\frac{ACC-randomACC}{1-randomACC}$$

(11) randomACC=p1p2+(1−p1)(1−p2)

(12) $$p1=\frac{TP+FN}{TP+TN+FP+FN}$$

(13) $$p1=\frac{TP+FN}{TP+TN+FP+FN}$$

In this study, classification error consists of two parts. The first is the model's error rate, while the second part is a confidence interval (CI). The second part is the probability of falling within this range. In CI, the constant indicates the table value against the chosen probability, and the *n* is the number of observations used when developing the model. Error rates for all classifiers have been measure with a 95% confidence interval. The categorical error is calculated with (14)–(16) (Brownlee, 2020).

(14) $$error=\;\frac{FP+FN}{TP+TN+FP+FN}$$

(15) $$CI=constant\sqrt{\frac{error\left(1-error\right)}{n}}$$

(16) ClassificationError=error±CI

All classification processes are carried out with k fold cross-validation, *k* = 10. Cross-validation separates the data set into ten separate training and test sets, and each time the classifier is trained and tested with different data (Arican & Polat, 2019). Software training data is 90%, and test data is 10%, automatically and randomly discriminating from both classes. In this direction, 174 of 1740 observations, being different for each floor, are used as training and the rest of the test.

### The obtained results with non-weighted features

The classification results of the MI dataset made without applying the weighting process for the kNN classifier are given in Table 4. Where EEG signal gave the highest result for the kNN classifier, it remained at 56.781%.

**Table 4:** Non-Weighted MI Dataset kNN Classification Results.

	ACC (%)	SENS	FPR	PRC	Kappa	Classification error
HbO	48.965	0.480	0.519	0.489	−0.020	0.510 ± 0.023
HbR	52.011	0.544	0.455	0.519	0.040	0.480 ± 0.023
EEG	56.781	0.565	0.434	0.568	0.135	0.432 ± 0.023
EEG+HbO	52.988	0.498	0.501	0.531	0.059	0.470 ± 0.023
EEG+HbR	52.586	0.537	0.462	0.525	0.051	0.474 ± 0.023
HbO+HbR	49.770	0.514	0.485	0.497	−0.004	0.502 ± 0.023

The classification results of the MI dataset made without applying the weighting process for the LDA classifier are given in Table 5. Similarly, the EEG signal gave the highest result for the LDA classifier; it remained 60.460%.

**Table 5:** Non-Weighted MI Dataset LDA Classification Results.

	ACC (%)	SENS	FPR	PRC	Kappa	Classification error
HbO	51.782	0.511	0.489	0.518	0.036	0.482 ± 0.023
HbR	54.253	0.551	0.449	0.542	0.085	0.457 ± 0.023
EEG	60.460	0.615	0.385	0.602	0.209	0.395 ± 0.023
EEG+HbO	50.977	0.544	0.456	0.509	0.020	0.490 ± 0.023
EEG+HbR	55.460	0.567	0.433	0.553	0.109	0.445 ± 0.023
HbO+HbR	52.816	0.572	0.428	0.526	0.056	0.472 ± 0.023

The classification results of the MI dataset made without applying the weighting process for the SVM classifier are given in Table 6. Again, the EEG signal gave the highest accuracy for the SVM classifier; it remained 60.402%.

**Table 6:** Non-Weighted MI Dataset SVM Classification Results.

	ACC (%)	SENS	FPR	PRC	Kappa	Classification error
HbO	52.184	0.549	0.451	0.521	0.044	0.478 ± 0.023
HbR	53.046	0.551	0.449	0.529	0.061	0.470 ± 0.023
EEG	60.402	0.603	0.397	0.604	0.208	0.396 ± 0.023
EEG+HbO	57.874	0.594	0.406	0.576	0.157	0.421 ± 0.023
EEG+HbR	59.943	0.632	0.368	0.593	0.199	0.401 ± 0.023
HbO+HbR	53.103	0.530	0.470	0.531	0.062	0.469 ± 0.023

The classification results of the MA dataset made without applying the weighting process are given in Table 7. Where EEG data gave the highest Accuracy rate for the kNN classifier, it remained at 62.701%.

**Table 7:** Non-Weighted MA Dataset kNN Classification Results.

	ACC (%)	SENS	FPR	PRC	Kappa	Classification error
HbO	56.724	0.556	0.444	0.569	0.134	0.433 ± 0.023
HbR	57.701	0.586	0.414	0.576	0.154	0.423 ± 0.023
EEG	62.701	0.663	0.337	0.618	0.254	0.373 ± 0.023
EEG+HbO	60.402	0.611	0.389	0.602	0.208	0.396 ± 0.023
EEG+HbR	61.552	0.675	0.325	0.603	0.231	0.384 ± 0.023
HbO+HbR	56.724	0.556	0.444	0.569	0.134	0.425 ± 0.023

The obtained classification results on the MA dataset without applying the weighting process are given in Table 8. HbO data gave the highest accuracy rate for the LDA classifier, it remained at 66.332%.

**Table 8:** Non-Weighted MA Dataset LDA Classification Results.

	ACC (%)	SENS	FPR	PRC	Kappa	Classification error
HbO	66.322	0.667	0.333	0.662	0.326	0.337 ± 0.022
HbR	63.966	0.656	0.344	0.635	0.279	0.360 ± 0.023
EEG	65.805	0.676	0.324	0.653	0.316	0.342 ± 0.022
EEG+HbO	59.885	0.653	0.347	0.589	0.198	0.401 ± 0.023
EEG+HbR	61.494	0.640	0.360	0.609	0.230	0.385 ± 0.023
HbO+HbR	63.391	0.725	0.275	0.613	0.268	0.366 ± 0.023

The classification results of the MA dataset made without applying the weighting process are given in Table 9. Where EEG+HbO data gave the highest Accuracy rate for the SVM classifier, it remained at 74.138%.

**Table 9:** Non-Weighted MA Dataset SVM Classification Results.

	ACC (%)	SENS	FPR	PRC	Kappa	Classification error
HbO	65.460	0.653	0.347	0.655	0.309	0.345 ± 0.022
HbR	64.770	0.647	0.353	0.648	0.295	0.352 ± 0.022
EEG	70.920	0.721	0.279	0.704	0.418	0.291 ± 0.021
EEG+HbO	74.138	0.743	0.257	0.741	0.483	0.259 ± 0.021
EEG+HbR	72.241	0.707	0.293	0.730	0.445	0.278 ± 0.021
HbO+HbR	70.287	0.694	0.306	0.706	0.406	0.297 ± 0.021

**Figure 5:**
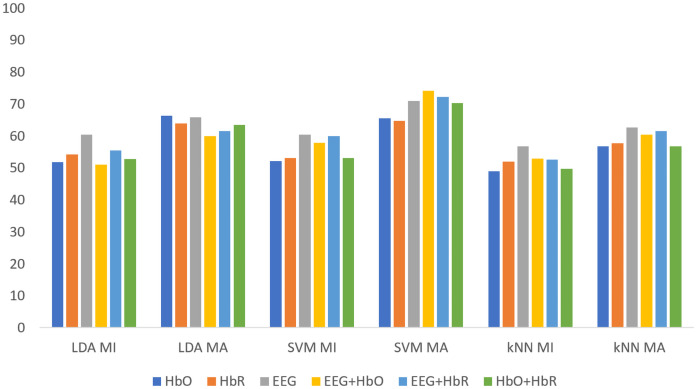
The comparison of non-weighted MI and MA tasks for all classifiers.

Figure 5 shows the classification results of the non-weighted MI and MA tasks for all three classifiers. Although the MA task gave higher accuracy than the MI task, it remained at fairly low levels.

### The obtained results with weighted features

**Figure 6:**
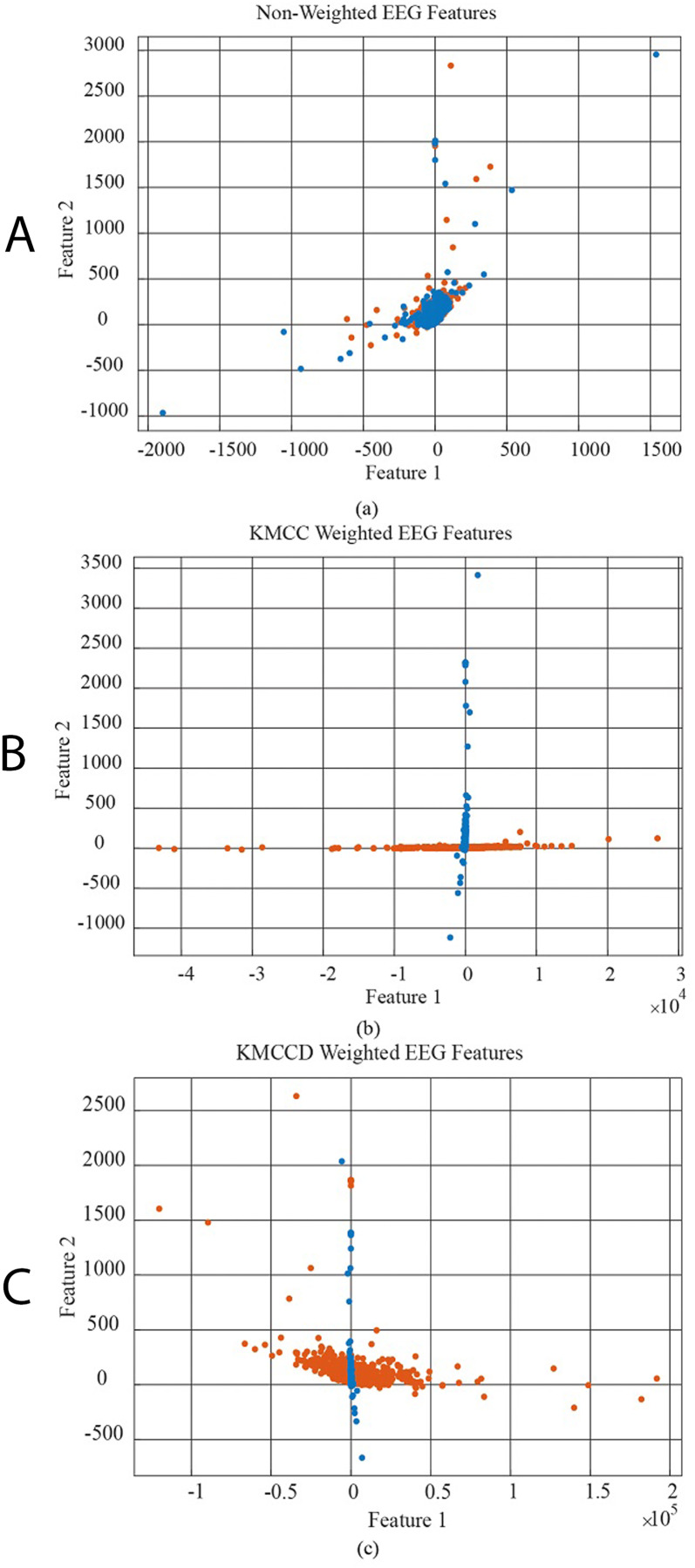
The comparison of 1st and 2nd features for the non-weighted and weighted data using MI tasks EEG features set, (A) non-weighted EEG features, (B) KMCC weighted EEG features, and (C) KMCCD weighted EEG features.

**Figure 7:**
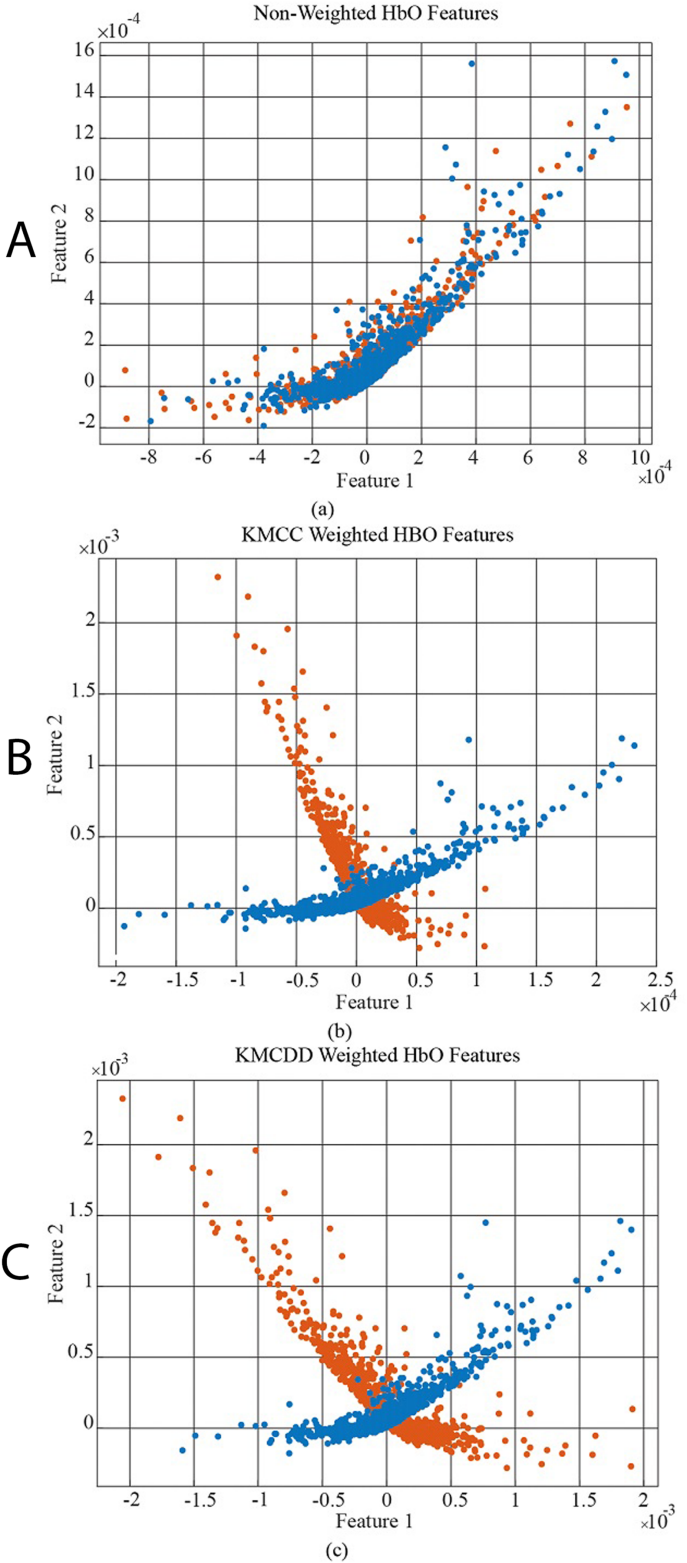
The comparison of 1st and 2nd features for the non-weighted and weighted data using MA tasks HbO features set, (A) Non-weighted HbO features, (B) KMCC weighted HbO features, and (C) KMCCD weighted HbO features.

In Fig. 6, the data distribution for feature 1 and feature 2 for the EEG+HbO signal belonging to the randomly selected MI task is given. Figure 6A shows the distribution of the unweighted data, Fig. 6B the KMCC-based weighted data distribution, and Fig. 6C the KMCCD-based weighted data distribution. The separation of weighted data can be insight. Figure 7 shows the comparison of 1st and 2nd features for non-weighted and weighted data of MA tasks HbO features set.

### k-means clustering centers based attribute weighting method (KMCC-based)

Table 10 shows the feature datasets’ results for the MI task for which KMCC based weighting algorithm is applied. The EEG+HBR data reaches an accuracy rate of 99.540%.

**Table 10:** KMCC Based Weighted MI Dataset kNN Classification Results.

	ACC (%)	SENS	FPR	PRC	Kappa	Classification error
HbO	95.920	0.962	0.038	0.957	0.918	0.041 ± 0.009
HbR	95.747	0.924	0.076	0.990	0.915	0.043 ± 0.009
EEG	95.172	0.922	0.078	0.980	0.903	0.048 ± 0.010
EEG+HbO	98.966	0.993	0.007	0.986	0.979	0.010 ± 0.005
EEG+HbR	99.540	0.997	0.003	0.994	0.991	0.005 ± 0.003
HbO+HbR	97.644	0.980	0.020	0.973	0.953	0.024 ± 0.007

Table 11 shows the KMCC based weighted MI dataset for LDA classification results. The EEG data give the highest value for the LDA classifier, the same as non-weighted EEG data for the LDA classifier with 97.816%.

**Table 11:** KMCC Based Weighted MI Dataset LDA Classification Results.

	ACC (%)	SENS	FPR	PRC	Kappa	Classification error
HbO	84.655	0.829	0.171	0.859	0.693	0.153 ± 0.017
HbR	88.908	0.853	0.147	0.919	0.778	0.111 ± 0.015
EEG	97.816	0.982	0.018	0.975	0.956	0.022 ± 0.007
EEG+HbO	91.667	0.994	0.006	0.861	0.833	0.083 ± 0.013
EEG+HbR	91.322	0.993	0.007	0.856	0.826	0.087 ± 0.013
HbO+HbR	70.575	0.618	0.382	0.749	0.411	0.294 ± 0.021

Table 12 shows the KMCC based weighted MI dataset for SVM classifier results. The fNIRS hybrid data give the highest value for the SVM classifier; it remained at 99.943%.

**Table 12:** KMCC Based Weighted MI Dataset SVM Classification Results.

	ACC (%)	SENS	FPR	PRC	Kappa	Classification error
HbO	97.816	0.999	0.001	0.959	0.956	0.022 ± 0.007
HbR	98.736	0.980	0.020	0.994	0.975	0.013 ± 0.005
EEG	97.816	0.979	0.021	0.977	0.956	0.022 ± 0.007
EEG+HbO	98.678	0.992	0.008	0.982	0.974	0.013 ± 0.005
EEG+HbR	98.621	0.997	0.003	0.976	0.972	0.014 ± 0.005
HbO+HbR	99.943	0.999	0.001	1.000	0.999	0.001 ± 0.001

Table 13 shows the KMCC based weighted MA dataset for the kNN classifier results. The fNIRS hybrid data give the highest value for EEG+HbR hybrid data and it remained at 98.793%.

**Table 13:** KMCC Based Weighted MA Dataset kNN Classification Results.

	ACC (%)	SENS	FPR	PRC	Kappa	Classification error
HbO	98.736	0.983	0.017	0.992	0.975	0.013 ± 0.005
HbR	89.138	0.783	0.217	1.000	0.783	0.109 ± 0.015
EEG	95.000	0.905	0.095	0.995	0.900	0.050 ± 0.010
EEG+HbO	98.563	0.971	0.029	1.000	0.971	0.014 ± 0.006
EEG+HbR	98.793	0.976	0.024	1.000	0.976	0.012 ± 0.005
HbO+HbR	94.425	0.889	0.111	1.000	0.889	0.056 ± 0.011

Table 14 shows the KMCC based weighted MA dataset for LDA classification results. The fNIRS hybrid data give the highest value for the LDA classifier; it remained at 97.356%.

**Table 14:** KMCC Based Weighted MA Dataset LDA Classification Results.

	ACC (%)	SENS	FPR	PRC	Kappa	Classification error
HbO	91.724	0.870	0.130	0.961	0.834	0.083 ± 0.013
HbR	88.621	0.832	0.168	0.933	0.772	0.114 ± 0.015
EEG	96.034	0.944	0.056	0.976	0.921	0.040 ± 0.009
EEG+HbO	97.356	0.985	0.015	0.963	0.947	0.026 ± 0.008
EEG+HbR	96.724	0.999	0.001	0.939	0.934	0.033 ± 0.008
HbO+HbR	76.782	0.746	0.254	0.780	0.536	0.232 ± 0.020

Table 15 shows the KMCC based weighted MA dataset for SVM classification results. The fNIRS hybrid data give the highest value for EEG+HbR hybrid data, and it remained at 99.655%.

**Table 15:** KMCC Based Weighted MA Dataset SVM Classification Results.

	ACC (%)	SENS	FPR	PRC	Kappa	Classification error
HbO	99.138	0.990	0.010	0.993	0.983	0.009 ± 0.004
HbR	97.989	0.960	0.040	1.000	0.960	0.020 ± 0.007
EEG	98.966	0.990	0.010	0.990	0.979	0.010 ± 0.005
EEG+HbO	99.425	0.999	0.001	0.990	0.989	0.006 ± 0.004
EEG+HbR	99.655	0.999	0.001	0.994	0.993	0.003 ± 0.003
HbO+HbR	94.598	0.902	0.098	0.989	0.892	0.054 ± 0.011

**Figure 8:**
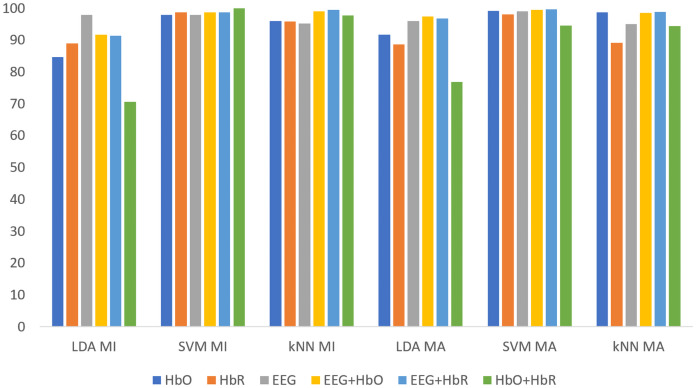
Comparison of KMCC-based weighted MI and MA tasks for all classifiers.

All classification results for MI and MA tasks are given comparatively in Fig. 8 for the KMCC-based weighted algorithm.

### k-means clustering centers difference based attribute weighting method (KMCCD-based)

Table 16 shows the feature datasets’ results for the MI task for which KMCCD based weighting algorithm is applied for the kNN classifier. The kNN classifier, which has the lowest accuracy rates in the non-weighted classification process, reached an accuracy rate of 99.655% (for EEG+HbR features) as in KMCC.

**Table 16:** KMCCD Based Weighted MI Dataset kNN Classification Results.

	ACC (%)	SENS	FPR	PRC	Kappa	Classification error
HbO	91.494	0.964	0.036	0.878	0.830	0.085 ± 0.013
HbR	90.920	0.972	0.028	0.863	0.818	0.091 ± 0.014
EEG	98.448	0.999	0.001	0.971	0.969	0.016 ± 0.006
EEG+HbO	99.540	0.998	0.002	0.993	0.991	0.005 ± 0.003
EEG+HbR	99.655	0.997	0.003	0.997	0.993	0.003 ± 0.003
HbO+HbR	94.598	0.930	0.070	0.961	0.892	0.054 ± 0.011

Table 17 shows the KMCCD based weighted MI dataset for LDA classification results. The EEG data give the highest value for the LDA classifier, and it remained at 96.724%.

**Table 17:** KMCCD Based Weighted MI Dataset LDA Classification Results.

	ACC (%)	SENS	FPR	PRC	Kappa	Classification error
HbO	74.253	0.720	0.280	0.754	0.485	0.257 ± 0.021
HbR	75.000	0.757	0.243	0.746	0.500	0.250 ± 0.020
EEG	96.724	0.992	0.008	0.945	0.934	0.033 ± 0.008
EEG+HbO	93.046	0.998	0.002	0.879	0.861	0.070 ± 0.012
EEG+HbR	93.103	0.997	0.003	0.881	0.862	0.069 ± 0.012
HbO+HbR	70.230	0.832	0.168	0.661	0.405	0.298 ± 0.021

Table 18 shows the KMCCD based weighted MI dataset for SVM classification results. The EEG + HbO data give the highest value for the SVM classifier, and it remained at 99.080%.

**Table 18:** KMCCD Based Weighted MI Dataset SVM Classification Results.

	ACC (%)	SENS	FPR	PRC	Kappa	Classification error
HbO	94.885	0.963	0.037	0.936	0.898	0.051 ± 0.010
HbR	96.437	0.943	0.057	0.986	0.929	0.036 ± 0.009
EEG	96.379	0.990	0.010	0.941	0.928	0.036 ± 0.009
EEG+HbO	99.080	0.993	0.007	0.989	0.982	0.009 ± 0.004
EEG+HbR	98.851	0.993	0.007	0.984	0.977	0.011 ± 0.005
HbO+HbR	81.954	0.871	0.129	0.790	0.639	0.180 ± 0.018

Table 19 shows the KMCCD based weighted MA dataset for kNN classification results. The EEG+HbR data give the highest value for the kNN classifier, and it remained at 99.885%.

**Table 19:** KMCCD Based Weighted MA Dataset kNN Classification Results.

	ACC (%)	SENS	FPR	PRC	Kappa	Classification error
HbO	92.471	0.955	0.045	0.900	0.849	0.075 ± 0.012
HbR	87.874	0.976	0.024	0.817	0.757	0.121 ± 0.015
EEG	93.966	0.886	0.114	0.992	0.879	0.060 ± 0.011
EEG+HbO	99.885	0.998	0.002	1.000	0.998	0.001 ± 0.002
EEG+HbR	99.770	0.995	0.005	1.000	0.995	0.002 ± 0.002
HbO+HbR	92.299	0.852	0.148	0.993	0.846	0.077 ± 0.013

Table 20 shows the KMCCD based weighted MA dataset for LDA classification results. The EEG + HbR data give the highest value for the LDA classifier, which remained at 98.793%.

**Table 20:** KMCCD Based Weighted MA Dataset LDA Classification Results.

	ACC (%)	SENS	FPR	PRC	Kappa	Classification error
HbO	78.851	0.782	0.218	0.793	0.577	0.211 ± 0.019
HbR	73.046	0.741	0.259	0.726	0.461	0.270 ± 0.021
EEG	94.713	0.962	0.038	0.934	0.894	0.053 ± 0.011
EEG+HbO	98.103	0.999	0.001	0.964	0.962	0.019 ± 0.006
EEG+HbR	98.793	0.998	0.002	0.979	0.976	0.012 ± 0.005
HbO+HbR	76.724	0.693	0.307	0.814	0.534	0.233 ± 0.020

Table 21 shows the KMCCD based weighted MA dataset for SVM classification results. The EEG+HbR data give the highest value for the LDA classifier, and it remained at 99.943%.

**Table 21:** KMCCD Based Weighted MA Dataset SVM Classification Results.

	ACC (%)	SENS	FPR	PRC	Kappa	Classification error
HbO	96.609	0.962	0.038	0.970	0.932	0.034 ± 0.009
HbR	97.816	0.979	0.021	0.977	0.956	0.022 ± 0.007
EEG	98.448	0.980	0.020	0.988	0.969	0.016 ± 0.006
EEG+HbO	99.713	0.997	0.003	0.998	0.994	0.003 ± 0.003
EEG+HbR	99.943	0.999	0.001	1.000	0.999	0.001 ± 0.001
HbO+HbR	85.690	0.801	0.199	0.902	0.714	0.143 ± 0.016

**Figure 9:**
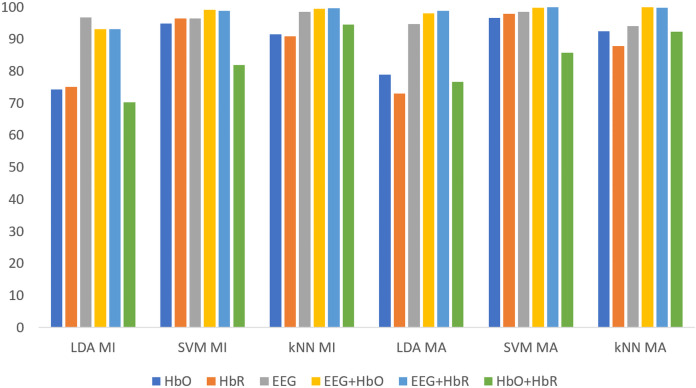
Comparison of KMCCD-based weighted MI and MA tasks for all classifiers.

All classification results for MI and MA tasks are given comparatively in Fig. 9 for the KMCCD-based weighted algorithm. Higher performances of EEG signal features and Hybrid features are seen.

The Classification Error-values are close to 0 here indicates that the rate of making an error in the label selected for externally entered data is low.

## Discussion

With the proposed model, an average 55% increase in accuracy is achieved in classification performances. However, an average performance increase of 65% is achieved in the MI task (85% for kNN). An increase in kNN classifier performance is obtained in both tasks. This shows that using the proposed fNIRS and EEG combined signals with the weighting method positively affects the system. Especially these results obtained with more basic classifiers such as kNN and SVM give hope for future studies

Table 22 lists the studies in the literature using the same data set. This study stands out with the simplicity of the model and the obtained accuracy rate compared to the literature studies. The results obtained show that the proposed model is a suitable method for hybrid BCI systems. When the accuracy rates, error amounts, and weighting algorithms are examined according to the classifier types specific to the MI and MA tasks, it is seen in the experimental results that the results are proportionally consistent with each other.

**Table 22:** Studies in the literature using the same data set.

Authors	Year	Task	Signal Type	Method	Classifier	ACC (%)	
Aydın	2020	MA MI	Hbo Hbr Hbo Hbr	SWR-SFS + SVM for MA SWR-SFS + LDA for MI)	SVM LDA	89 86 78 77	(Aydin, 2020)
Jiang et.al.	2019	MA MI	Hybrid	Indıpendent Decision Path Fusion	PCA+LDA	91 78	(Jiang et al., 2019)
Ergun et.al	2018	MA MI	Hbo Hbr Hbo Hbr	Features extraction by Katz fractal dimension	Knn	83 85 72 70	(Ergün & Aydemir, 2018)
Shin et.al	2017	MA MI	Hybrid	CSP+Mean Value and Avarage Slope Features	LDA	91 78	(Shin et al., 2017)
Proposed Method		MA	Hybrid	KMCC KMCCD	kNN/SVM kNN/LDA/SVM	99	
		MI		KMCC KMCCD	kNN/SVM kNN/LDA/SVM	99	

Table 22. The conducted works using MI and MA tasks with the other state of the art methods in the literature

## Conclusions

BCI systems will become more applicable with the measurements of the brain, which are expected to become easier in the developing and progressive process. There are some difficulties in the implementation of the systems designed at this stage. Although it gives relatively good results, especially in systems that require more data and more capacity, such as deep learning, it makes application conditions difficult. For this reason, improving traditional techniques like the proposed method will bring the applicability of BCI systems one step forward. The studies will be made more applicable by transferring the theoretical calculations to applied studies and compacting the measurement systems. The results show that the proposed method increases classifiers' performance, offering less processing power and ease of application. In the future, the new studies could be carried out by combining the k-means clustering center-based weighted hybrid BCI method with deep learning architectures.

## Additional Information and Declarations

### Data Availability

The following information was supplied regarding data availability: The source code is available at GitHub: https://github.com/kpolat14/eeg-and-fnirs-matlab-code. The data are publicly accessible at the Open access dataset for simultaneous EEG and NIRS brain-computerinterface (BCI). The site requires registration and a brief description of your research area for statistical purposes. This data was made available for peer review. LINK: http://doc.ml.tu-berlin.de/hBCI/.
